# The complete mitochondrial genome of click beetle *Chiagosnius sulcicollis* (Coleoptera: Elateridae) and phylogenetic analysis

**DOI:** 10.1080/23802359.2019.1627936

**Published:** 2019-07-12

**Authors:** Yang Wang, Xuan Wang, Yang Liu

**Affiliations:** a College of Biology Pharmacy and Food Engineering, Shangluo University, Shangluo, China;; b Shaanxi provincial institute of forestry investigation and planning, Xi’an, China;; c Key Laboratory of Resource Biology and Biotechnology in Western China (Ministry of Education) and College of Life Science, Northwest University, Xi’an, China

**Keywords:** Insecta, molecular phylogeny, mitochondrial DNA, genetic diversity

## Abstract

In this study, we determined the complete mitochondrial genome sequence of click beetle *Chiagosnius sulcicollis* (Candeze, 1878) (GenBank accession no. MK792747) using next-generation sequencing (NGS) method. The mitogenome is 15,848 bp in length, consisting of 13 protein-coding genes (PCGs), 2 ribosomal RNA genes, 22 transfer RNA genes, and 1 non-coding control region. The overall nucleotide composition was 41.6% A, 31.5% T, 16.6% C, and 10.3% G, with 73.1% AT, respectively. The gene arrangement is consistent with the typical insect mitochondrial genome. Phylogenetic analysis revealed that *C. sulcicollis* clustered into a clade with *Melanotus villosus* with high bootstrap support.

Elateridae commonly known as click beetles due to a unique and well-known startling defence mechanism named ‘clicking’ contains approximately 10,000 species (Johnson 2002). *Chiagosnius sulcicollis*, with yellowish-brown vertical stripe in the middle of the fore wing, mainly distributed in southern China and Southeast Asia (Jiang and Wang 1999). Here, we determined the complete mitochondrial genome of *C. sulcicollis*, which is the first mitochondrial genome sequenced to date in the genus of *Chiagosnius*.

The sampled specimen was collected from Jianfengling National Nature Reserve, Hainan, China (18°44′ N, 108°54′ E) in May, 2018. The specimen was stored in the Entomological specimen room of Shangluo University. The complete mitochondrial DNA sequence was determined by Illumina HiSeq 2500 Sequencing System (Illumina, San Diego, CA). In total, 5.2 Gb raw reads were obtained, quality-trimmed, and assembled using MITObim v1.7 (Hahn et al. 2013).

The complete mitochondrial genome of *C. sulcicollis* was 15,848 bp in total length and deposited in GenBank database with an accession number MK792747. The overall base composition was 41.6% A, 31.5% T, 16.6% C, and 10.3% G, with an A + T ratio of 73.1%. The full mitochondrial genome contains 13 protein-coding genes (PCGs), 22 transfer RNAs (tRNAs), two ribosomal RNAs (rRNAs) and a putative control region (CR). The gene arrangement of *C. sulcicollis* is found to be similar to most insect mitochondrial genomes (Wolstenholme 1992). Most PCGs of *C. sulcicollis* have the conventional start codon for invertebrate mitochondrial PCGs (ATN), with the exception of *nad1* (TTG) and *cox1* (AAT), as the asparagine (AAT or AAC) are proposed to be the start codon for *cox1* in suborder Polyphaga (Sheffield et al. 2008). Most of the PCGs terminate with the stop codon TAA or TAG, whereas *cox2*, *cox3* and *nad5* end with the incomplete codon T. Nine protein-coding genes are encoded on the majority strand (J-strand) and four (*nad5*, *nad4*, *nad4L*, and *nad1*) on the minority strand (N-strand). All 22 tRNA genes can be folded into the typical cloverleaf structure except for *trnS1*, in which the dihydrouridine (DHU) arm cannot form a stable stem-loop structure but a simple loop. Two rRNA genes (*rrnL* and *rrnS*) locate at *trnL1*/*trnV* and *trnV*/control regions, respectively, and both rRNA genes are encoded on the N-strand. The lengths of the two rRNA genes (*rrnL* and *rrnS*) in *C. sulcicollis* are about 1283 and 738 bp, with the A + T contents of 78.1 and 75.9%, respectively. The length of the control region is 1235 bp, with the AT content of this region being ∼81.3%.

The phylogenetic tree was constructed using the maximum-likelihood method through raxmlGUI 1.5 (Silvestro and Michalak 2012). Results showed that the family Elateridae is monophyletic and *C. sulcicollis* is sister to *Melanotus villosus* (Figure 1), which was consistent with the previous studies (Douglas 2011; Lin et al. 2018; Meng et al. 2018). In conclusion, we obtained and described the complete mitochondrial genome of *C. sulcicollis*, which constitute a valuable and useful resource for population genetic study and identification efforts on this species.

**Figure 1.**
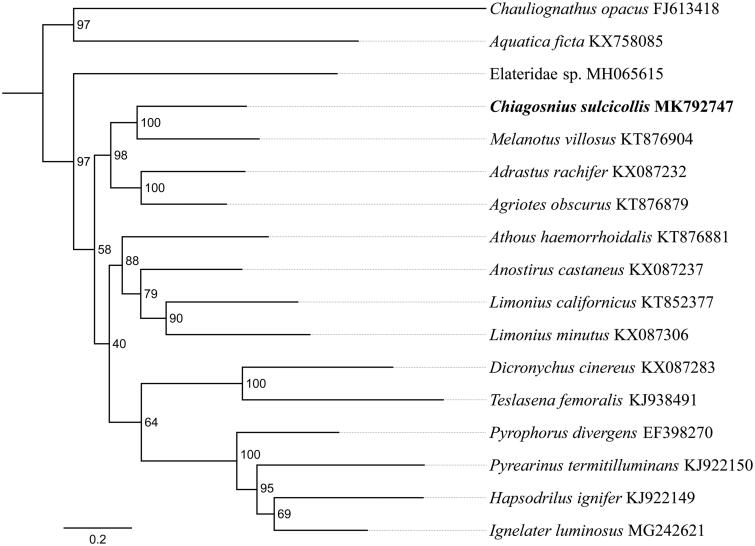
Phylogenetic relationships based on the 13 mitochondrial protein-coding genes sequences inferred from RaxML. Numbers on branches are Bootstrap values (BV).
